# Comparison of Surgical Outcomes Between Single-Site Laparoscopic Adenomyomectomy and Fundusectomy: Experience from a Single Center

**DOI:** 10.3390/jcm14124044

**Published:** 2025-06-07

**Authors:** Jae Hyeok Jeong, Yoo Ri Kim, Bo Ram Kim, Ji Won Lee, Seung Yeon Oh, Jeong Hye Yun, Myoung Seok Han, Yong Jung Song, Ki Hyung Kim

**Affiliations:** 1 Department of Obstetrics and Gynecology, Yeonje Ilsin Hospital, Busan 04991, Republic of Korea; drsoon@hanmail.net (J.H.J.); hanbie79@naver.com (Y.R.K.); ukino327@gmail.com (B.R.K.); angel98@daum.net (J.W.L.); syoh090290@gmail.com (S.Y.O.); dskyhigh@naver.com (J.H.Y.); hmsobgy@gmail.com (M.S.H.); 2 Department of Obstetrics and Gynecology, School of Medicine, Pusan National University, Busan 46241, Republic of Korea; 3 Research Institute for Convergence of Biomedical Science and Technology, Pusan National University Yangsan Hospital, Yangsan 50612, Republic of Korea; 4 Biomedical Research Institute, Pusan National University Hospital, Busan 49241, Republic of Korea

**Keywords:** single-site, laparoscopic adenomyomectomy, fundusectomy, adenomyosis

## Abstract

**Objective**: This study aimed to introduce clinical experience using a new surgical technique, single-port access laparoscopic fundusectomy, a more efficient uterus-sparing surgical method for adenomyosis in patients with no plans for pregnancy. **Methods**: We performed single-port access laparoscopic myomectomy in 141 patients and single-port access laparoscopic fundusectomy in 124 patients and compared the surgical outcomes. **Results**: Significant differences in surgical outcomes were observed between the two operating methods. Operative time was 158.19 min (±42.93) in the fundusectomy group and 179.11 min (±56.95) in the adenomyomectomy group (*p* = 0.001). Estimated blood loss was 175.04 mL (±142.76) in the fundusectomy group and 347.97 mL (±409.78) in the adenomyomectomy group (*p* = 0.000). The fundusectomy group showed smaller uterus size and volume, and lower postoperative CA125 levels than the adenomyomectomy group for 24 months (*p* = 0.000). **Conclusions**: This study suggests that single-port access laparoscopic fundusectomy is more effective in terms of operative time, estimated blood loss, and postoperative CA125 decrease than single-port access laparoscopic adenomyomectomy.

## 1. Introduction

Adenomyosis is a serious disease in women [1]. Generally, in women who want to fall pregnant in the future, oral contraceptives (OCs) or levonorgestrel-releasing intrauterine system (LNG-IUS) are used to control symptoms such as menorrhagia and dysmenorrhea. When pregnancy is desired and in cases where pregnancy is difficult or adenomyosis symptoms are severe, adenomyomectomy is performed. Hysterectomy is recommended in women who do not want to fall pregnant. However, in women who desire to fall pregnant, laparoscopic adenomyomectomy requires sufficient assurance of the uterine wall and the degree of adenomyosis removal, making the surgery challenging, with a relatively long operation time [2]. In addition, due to the high possibility of recurrence after adenomyomectomy, medication or reoperation may be necessary; hysterectomy is fundamentally recommended for women who do not want to fall pregnant [3,4]. However, post-hysterectomy depression in women is reported to lower the quality of life significantly, and the psychological relief provided to women by the uterus is not evaluated as insignificant [5,6]. Fundusectomy is the surgical removal of the fundus, the upper portion of an organ, such as the uterus or the stomach. The uterine fundosectomy has been less studied than the one in the stomach. To date, there are few studies on fundusectomy in the treatment of uterine adenomyosis. Uterine fundusectomy may be performed in women who want to preserve their uterus, which leads to a positive impact on patient satisfaction. Further, uterine preservation may also have a psychologically positive effect on a woman’s quality of life [7]. Thus, the current study aimed to compare the surgical outcomes of two different uterine sparing treatments for adenomyosis in patients with no plans for pregnancy.

## 2. Materials and Methods

### 2.1. Inclusion and Exclusion Criteria

This study retrospectively reviewed the clinical records of 265 patients who underwent single-port laparoscopic adenomyomectomy or fundusectomy (the surgical removal of the fundus, the upper portion of the uterus) at our hospital between January 2021 and September 2024. All surgeries were performed by two surgeons. Inclusion criteria consisted of premenopausal women with significant symptoms and candidates for hysterectomy due to adenomyosis. Women with a history of cancer, hereditary cancer, and patients who were candidates for surgery due to a malignant pathology were excluded.

### 2.2. Data Items and Outcomes

Patient information was collected from admission records, outpatient records, and surgery records, and included patient age, body mass index, parity, surgical history, preoperative uterine maximum diameter and uterine volume, concurrent surgeries such as ovarian surgery, operation time, degree of adhesion, presence of endometriosis, size and weight of the removed adenomyosis, intraoperative blood loss, preoperative and postoperative hemoglobin changes, preoperative and postoperative hematocrit change, changes in CA125 levels, presence of transfusion, presence of complications, postoperative hospital stay duration, uterine size and volume at 1 month, 3 months, 6 months, 1 year, and 2 years postoperatively, and presence of reoperation due to recurrent adenomyosis. The operative time was measured from the start of the incision below the navel to the end of skin closure, and the preoperative and postoperative hemoglobin and hematocrit changes were defined as the differences between the last preoperative measurement and the first postoperative measurement. The amount of bleeding during surgery was calculated by the anesthesiologist and their assistant as the difference between the total suction and irrigation amount. Complications were defined as conditions requiring additional treatment due to surgery, including fever over 5 days, genitourinary system issues, organ injury, intra-abdominal hematoma, transfusion, and reoperation. Postoperative hospital stay duration was classified as 3 days or less and extended due to complications and patient symptoms into 6–8 days, 9–12 days, and ≥13 days. The study was performed in accordance with the ethical standards described in the Declaration of Helsinki and was approved beforehand by the Institutional Review Board (IRB, YJISH-24-20007) of this institution, which waived the requirement for written informed consent because of the retrospective nature of the study.

### 2.3. Patient Characteristics

The average age of the 265 patients was 42.82 (±5.84) years (Table 1), and the average BMI was 22.98 (±3.53). There were 97 patients (36.6%) with previous abdominal operative history: 22 cases (8.3%) of one-time cesarean section, 29 cases (10.9%) of two or more cesarean sections, 15 cases (5.7%) of previous myomectomy, 22 cases (8.3%) of adnexal surgery, 4 cases (1.5%) of appendectomy, and 1 case of other abdominal surgery. Sixty-three patients (23.8%) also had endometriosis of the ovaries. The average size of the uterine longitudinal axis was 10.17 cm (±7.33), the expected uterine weight was 300.19 g (±175.85), and the preoperative CA125 was 92.00 U/mL (±128.82). The indications for surgery from the patient’s point of view, were menorrhagia in 114 cases (43.0%), severe dysmenorrhea in 94 cases (35.5%), increase in the size of the uterus in 9 cases (3.4%), pelvic pain in 17 cases (6.4%), frequent urination in 9 cases (3.4%), concurrent ovarian tumor in 3 cases (1.1%), a large mass in the abdomen in 1 case (0.4%), and concurrent myoma in 17 cases (6.4%). There were no cases in which the surgery was converted to a multi-port laparoscopic technique or laparotomy.

### 2.4. Surgical Method

All patients received prophylactic antibiotics 30 min before surgery, which is generally required for surgical preparation. General anesthesia was induced in the lithotomy position, and after cervical dilation, a uterine manipulator (Koh Colpotomizer, Colpo-Pneumo Occluder, RUMI; Cooper Surgical, Trumbull, CT, USA) was inserted. The single-site laparoscopic technique we used is as follows: first, we made a 2–2.5 cm vertical incision above the navel, reaching the fascia, and after confirming the presence of adhesion, an EASY-One Port (C-BAA, BJ Co., Ltd., Daegu, Republic of Korea) was inserted. After insufflation of carbon dioxide gas at 12 mmHg into the abdominal cavity, a 5 mm, 30 cm laparoscope (Telescope, Karl Storz, Tuttlingen, Germany) was inserted to explore the location and size of the adenomyosis. Grasping instruments, mainly linear instruments of 43 cm length similar to a general laparoscope, were used, but depending on the case, some instruments that allow bending were also used. Before removing the adenomyosis, vasopressin 20IU was diluted with saline at a ratio of 1:80 and injected into the uterus, and the adenomyosis was removed using monopolar and bipolar coagulative forceps. In all patients, the resected adenomyosis was removed with a scalpel through a single-port, without using a power morcellator. In cases where malignant transformation of the uterus was suspected or a large number of fragments were expected due to excision, the resected adenomyosis was removed via scalpel morcellation and placed in a vinyl bag. After removing the adenomyosis, the wound was sutured using a continuous suture method in two layers; in the case of fundusectomy, the uterine endometrium was sutured in a single layer, and the myometrium was sutured with a two layers or overriding method. The suture material used was Monosyn 1-0. The excised adenomyosis tissue was extracted through the navel using a scalpel. A drainage tube was inserted through the incision in the navel where the surgery was performed, and if necessary, the postoperative uterotonic agent, carbetocin (Duratocin inj. 100 ug/mL) was used.

### 2.5. Statistical Analysis

Statistical analysis of the research results was performed using SPSS ver. 18.0 (SPSS Inc., Chicago, IL, USA). The Student *t*-test, Mann-Whitney U test, and Chi-square test were used, and a *p*-value < 0.05 was considered statistically significant.

## 3. Results

Patients in the fundusectomy group tended to be generally older (45.06 years vs. 40.27 years) and had larger uterine weights (332.55 g vs. 263.09 g) compared with patients undergoing adenomyomectomy (Table 2). Fundusectomy was performed in 141 cases. There were 57 cases (40.4%) with a history of abdominal surgery. Concurrent endometriosis was suspected preoperatively in 22 (15.6%) patient. During surgery, severe endometriosis was confirmed in 35 (24.8%) patients. In adenomyomectomy group, there were 40 cases (32.3%) with previous abdominal operative history, and there were 41 cases (33.1%) where concurrent endometriosis was suspected. Severe endometriosis was confirmed in 37 (29.8%) patients.

**Figure 1:**
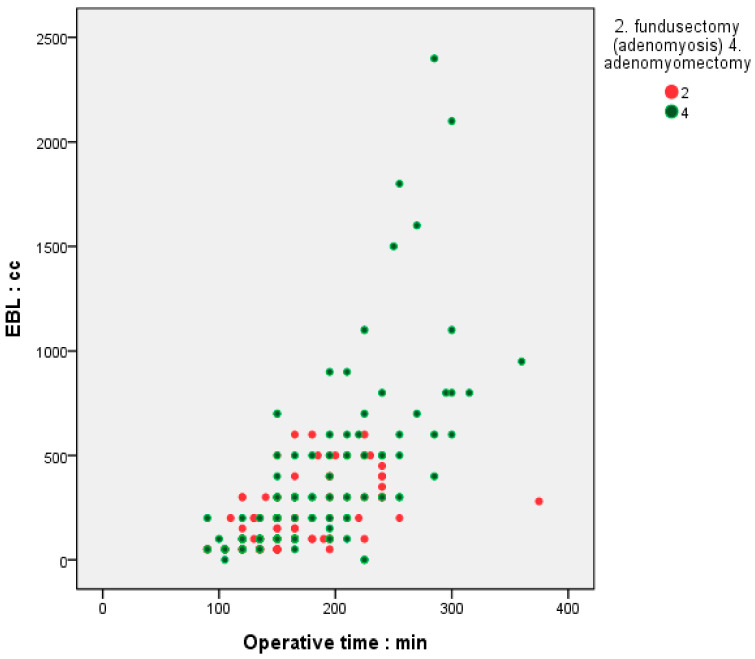
Comparison of operative time and blood loss between single-port laparoscopic fundusectomy and adenomyomectomy.

Surgical outcomes based on the methods are shown (Table 3). In fundusectomy, the average size and weight of resected adenomyosis were 5.90 cm (±1.94) and 275.73 g (±173.56), respectively. In adenomyomectomy, the size and weight of resected adenomyosis were 5.46 cm (±1.78) and 94.34 g (±94.14), respectively, showing a statistically significant difference between the groups. Fundusectomy was associated with a shorter operative time (158.19 min vs. 179.11 min) and less blood loss (175.04 mL vs. 347.97 mL) than adenomyomectomy, as shown in Table 3. The correlation between operation time and estimated blood loss showed a significant relationship, with a Pearson’s correlation coefficient of 0.644 and a Spearman’s correlation coefficient of 0.703 (Figure 1). Fundusectomy was associated with a smaller decrease in hemoglobin and hematocrit and a lower transfusion rate than adenomyomectomy (Table 3). Most patients had a typical postoperative hospital stay less than 3 days.

**Figure 2:**
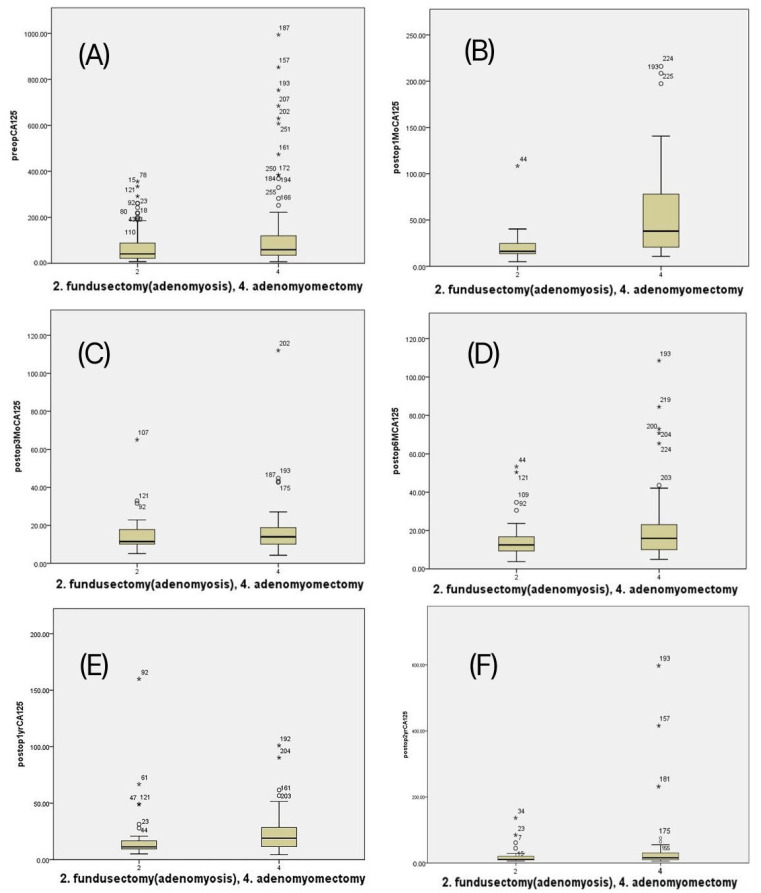
Change of CA125 in patients undergone with single-site laparoscopic fundusectomy vs. adenomyomectomy. (**A**) preoperative, (**B**) at 1 Mo, (**C**) at 3 Mo, (**D**) at 6 Mo, (**E**) at 12 Mo, and (**F**) at 24 Mo.

During the 2-year postoperative follow-up, uterine size, volume, and CA125 levels were monitored. Fundusectomy led to a consistent reduction in uterine size and CA125 levels, which remained within the normal range throughout the 24-month follow-up (Table 4, Figure 2). In contrast, the adenomyomectomy group showed a gradual increase in these parameters over time. Only patients in the adenomyomectomy group required reoperation during the follow-up period (Table 4). Fundusectomy resulted in a smaller uterine size and volume and lower CA125 levels after surgery than adenomyomectomy.

## 4. Discussion

**Figure 3:**
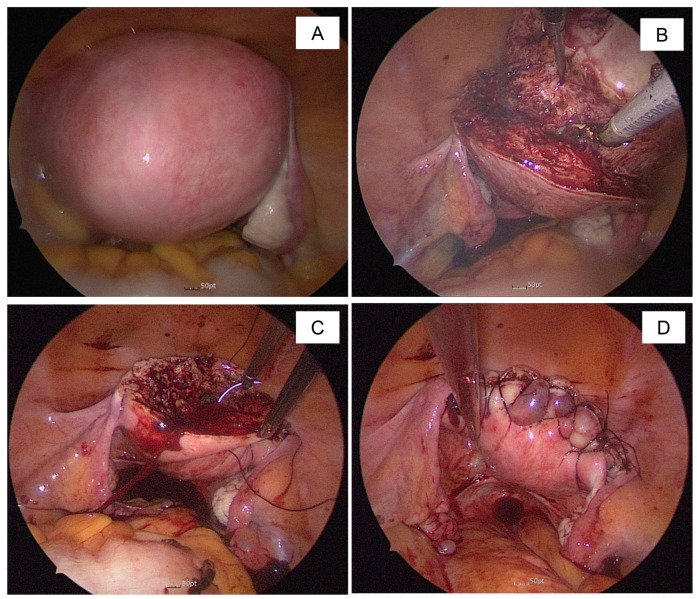
Intraoperative views in single-site laparoscopic fundusectomy (**A**–**D**). (**A**) Preoperative; (**B**) Fundusectomy; (**C**) Suturing; (**D**) After suture.

Adenomyosis is a condition frequently observed in women of childbearing age, where endometrial glands and stroma infiltrate intramurally, causing severe dysmenorrhea and menorrhagia, significantly affecting women’s lives. Conservative treatments for adenomyosis include medical treatments such as NSAIDs, levonorgestrel-releasing intrauterine device (LNG-IUS), oral contraceptives (OCs), GnRHa, and various available treatments, such as uterine artery embolization (UAE), high-intensity focused ultrasound (HIFU), and radiofrequency ablation. Surgical treatments include hysterectomy, adenomyomectomy, and cytoreductive surgery [8,9,10]. Generally, for women who do not need to preserve fertility, hysterectomy is recommended, but for those who wish to avoid psychological issues after hysterectomy, such as depression or other problems, uterine-sparing surgical interventions have been developed [11,12,13,14]. However, if medical treatment is not provided after surgery, recurrence is common, and reoperation is often required due to symptom recurrence [15,16]. To prevent such recurrence, sufficient cytoreductive surgery for adenomyosis is recommended [17]. However, cytoreductive surgery for adenomyosis is challenging, especially in securing a flap for uterine reconstruction after adenomyosis resection. This study shows that fundusectomy is a possible solution [7,18,19]. Surgical conservative treatments of adenomyosis are very effective in ameliorating symptoms. However, few studies have compared the utility of fundusectomy technique to the limitations of current procedures. The advantage of fundusectomy is that it can sufficiently remove adenomyosis tissue, has a short operation time and low blood loss, and can adequately secure a double flap for uterine reconstruction, proving its effectiveness [20]. The term fundusectomy is commonly used for fundectomy when removing only the upper part of the stomach in stomach surgery; however, from a gynecologic perspective, fundusectomy is considered a more appropriate term. To date, few studies have been published on the use of fundusectomy in the treatment of uterine adenomyosis. Saremi et al. proposed surgical fundectomy in patients who were candidates for hysterectomy for benign indications [7]. Fundusectomy is a surgical method that removes adenomyosis located in the upper part of the uterine fundus, reduces the adenomyosis volume of the lower uterine segment [7], and creates an anterior and posterior flap with excision of the wedge type to shorten the operation time and reduce blood loss (Figure 3).

In this study, the average size and weight of the removed adenomyosis in fundusectomy were 5.90 cm (±1.94) and 275.73 g (±173.56), respectively, and in adenomyomectomy, the average size and weight were 5.46 cm (±1.78) and 94.34 g (±94.14), respectively, confirming significant differences and indicating that sufficient adenomyosis removal was achieved in fundusectomy. In fundusectomy, the average operation time was 158.19 min (±42.93) and the estimated blood loss was 175.04 mL (±142.76), while in adenomyomectomy, the average operation time was 179.11 min (±56.95) and the estimated blood loss was 347.97 mL (±409.78), showing that fundusectomy had a significantly shorter operation time and less estimated blood loss. Postoperative hemoglobin drop was also significantly lower in fundusectomy, with an average of 1.46 g/dl (±0.81) compared with 1.79 g/dl (±1.29) in adenomyomectomy, indicating a meaningful reduction in postoperative hemoglobin drop in fundusectomy.

**Figure 4:**
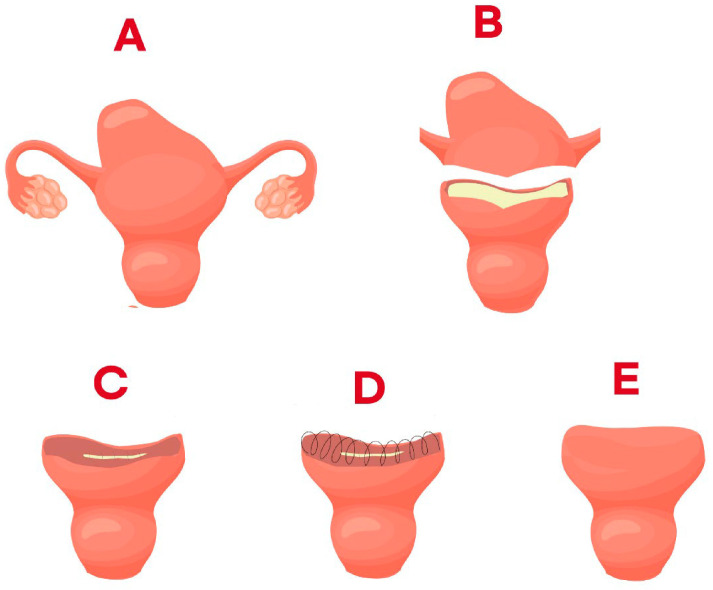
Operation procedures in single-site laparoscopic fundusectomy (**A**–**E**).Operation procedures in single-site laparoscopic fundusectomy in order from A to E.

The method for performing the fundusectomy procedure is illustrated in Figure 4. The adenomyotic lesion, including the uterine fundus, is resected using a monopolar cautery or energy device. The resection is performed in a way that leaves a flap that can cover the remaining uterine body and ensures that the uterine insertion area of both salpinx is not left behind. As much of the area with adenomyosis as possible was removed, and as much of the area with normal myometrium was left. The flap on both sides did not necessarily need to be similar, and the plane was sutured by overriding the smaller remaining flap with the larger remaining flap, designing it to be removed. The remaining endometrium was continuously sutured using Monosyn 1-0, and the remaining myomectium of the uterine body was continuously sutured by folding it in an overriding double-flap method. At this time, the suture can be performed using an in–out or an out–in method, and there is no association with the surgical outcome. However, if the remaining adenomyosis tissue is sutured, healing can be difficult; therefore, it is folded with a sufficiently extended flap of normal myomectrium. If this part is folded, it becomes the shape of the uterine fundus. The endometrium is continuously folded; if the remained myometrium is folded once more to take the form of a double fold, it can reduce the risk of uterine perforation during procedures such as dilatation and curettage (D&C) in the future, and it is thought to be able to prevent the recurrence of adenomyosis.

**Figure 5:**
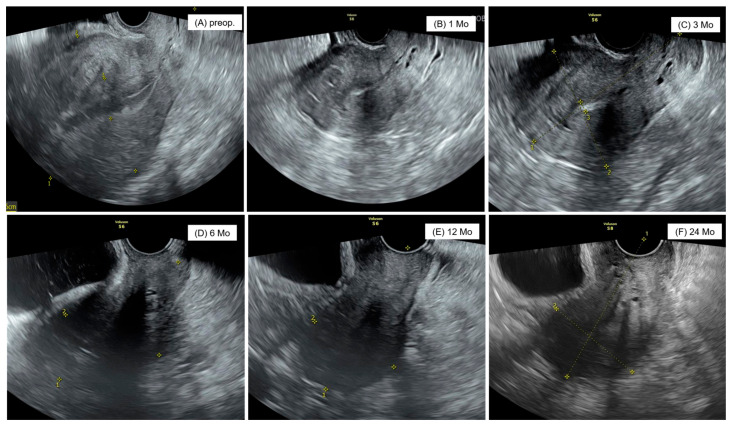
Postoperative ultrasound image of uterus in patient undergone with single-site laparoscopic fundusectomy. (**A**) preoperative, (**B**) at 1 Mo, (**C**) at 3 Mo, (**D**) at 6 Mo, (**E**) at 12 Mo, and (**F**) at 24 Mo.

The shape of the uterus was well maintained in the consecutive ultrasound examination after fundusectomy, and symptoms such as menorrhagia and dysmenorrhea and CA125 levels showed a significant decrease. During the two-year follow-up, there were no cases in which the uterus became larger or the clinical symptoms worsened, and no other complications due to surgery were observed (Figure 5). In adenomyomectomy, the uterine volume increased again 6 months after surgery, and CA125 levels increased again 24 months after surgery, with severe symptoms in eight patients, leading to reoperation. Among them, four patients underwent hysterectomy, and four patients underwent fundusectomy. One strength of the current study is that it focused on a new surgical technique, single-port access laparoscopic fundusectomy, uterus-sparing surgical method for adenomyosis in patients with no plans for pregnancy that has not been often addressed previously. However, this study had several limitations. First, the single-center, retrospective design of the present study is a notable limitation. Second, the sample sizes were insufficient to generalize our findings. Third, type, extension, and localization of adenomyosis was not specified. We acknowledge these limitations as opportunities for improvement in future multicenter prospective studies.

## 5. Conclusions

This retrospective study showed that single-port access laparoscopic fundusectomy is more effective in terms of operative time, estimated blood loss, and postoperative CA125 level decrease than single-port access laparoscopic adenomyomectomy. The patients who underwent adenomyomectomy had higher preoperative CA125 levels (at least double) and a two times higher likelihood of concurrent endometriosis, hinting at a more extensive underlying disease and a more challenging surgery than fundusectomy, thus leading to longer recovery times. Fundusectomy is considered a useful cytoreductive surgical method for women with adenomyosis who do not want to fall pregnant and want to preserve the uterus. During the two-year follow-up, there was almost no risk of recurrence, and it is considered an effective surgical method that does not require additional medical treatment after surgery. Our findings will have an impact on practice in the treatment of adenomyosis and will help with patient selection and appropriate counseling. Further prospective studies are necessary to validate the surgical outcomes of fundusectomy in adenomyosis patients.

## Figures and Tables

**Table 1:** Clinical characteristics of 265 patients.

Characteristics	Mean ± SD No of Patients (%)
Age (years)	42.82 ± 5.84
BMI (kg/m^2^)	22.98 ± 3.53
Previous abdominal operative history	97 (36.6%)
Number of previous abdominal operative history	0.53 ± 0.80
Previous operation type	
CS	22 (8.3%)
Twice or more CS	29 (10.9%)
Myomectomy	15 (5.7%)
Adnexa operation	22 (8.3%)
Appendectomy	4 (1.5%)
Diagnostic laparoscopy	0
Cholecystectomy	4 (1.5%)
Other abdominal surgery	1 (0.4%)
Preoperative longitudinal length of uterus (cm)	10.17 ± 7.33
Volume of uterus (gm)	300.19 ± 175.85
CA125 (U/mL)	92.00 ± 128.82
Combined endometriosis	63 (23.8%)
Operative indication	
Menorrhagia	114 (43.0%)
Dysmenorrhea	94 (35.5%)
Enlarged size	9 (3.4%)
Pelvic pain	17 (6.4%)
Urinary frequency	9 (3.4%)
Combined ovarian mass	3 (1.1%)
Palpable abdominal huge mass	1 (0.4%)
Combined myoma	17 (6.4%)

**Table 2:** Preoperative assessment for clinical characteristics of patients according to single-site laparoscopic fundusectomy vs. adenomyomectomy.

Clinical Outcome	Mean ± SD, No. of Patients (%)		
	Fundusectomy	Adenomyomectomy	*p*-Value
Number of patients	141	124	
Age (years)	45.06 ± 4.60	40.27 ± 6.07	0.00
20–29	0	8 (6.5%)	
30–39	18 (12.8%)	44 (35.5%)	
40–49	101 (71.6%)	69 (55.6%)	
>50	22 (15.6%)	3 (2.4%)	
Preoperative longitudinal length of uterus (cm)	10.04 ± 1.90	10.32 ± 10.56	0.76
Preoperative weight of uterus (gm)	332.55 ± 193.97	263.09 ± 144.53	0.00
Previous abdominal operative history	57(40.4%)	40(32.3%)	0.20
Number of previous abdominal operative history	0.58 ± 0.85	0.47 ± 0.74	0.25
Previous operation type			
CS	2 (5.7%)	9 (3.9%)	
Twice CS	2 (5.7%)	2 (0.9%)	
Myomectomy	7 (20.0%)	10 (4.4%)	
Adnexa operation	2 (5.7%)	7 (3.1%)	
Appendectomy	0	7 (3.1%)	
Diagnostic laparoscopy		7 (3.1%)	
Cholecystectomy	1 (2.9%)	2 (0.9%)	
Other abdominal surgery	1 (2.9%)	1 (0.4%)	
Combined with myoma	6 (4.3%)	11 (8.9%)	
Preoperative CA125 (U/mL)	68.37 ± 70.26	119.55 ± 170.03	0.00
Combined with predicted endometriosis	22 (15.6%)	41 (33.1%)	0.00

**Table 3:** Comparison of postoperative factors according to single-site laparoscopic fundusectomy vs. adenomyomectomy.

Clinical Outcome	Mean ± SD, No. of Patients (%)		
	Fundusectomy	Adenomyomectomy	*p*-Value
Largest size of resected adenomyosis (cm)	5.90 ± 1.94	5.46 ± 1.78	0.05
Weight of resected adenomyosis (gm)	275.73 ± 173.56	94.34 ± 94.14	0.00
Operation time (min)	158.19 ± 42.93	179.11 ± 56.95	0.00
Estimated blood loss (mL)	175.04 ± 142.76	347.97 ± 409.78	0.00
Change in hemoglobin (g/dL)	1.46 ± 0.81	1.79 ± 1.29	0.13
Change in hematocrit (%)	4.77 ± 2.74	4.83 ± 3.19	0.87
Transfusion	9 (6.4%)	19 (15.3%)	0.01
Pelvic adhesion due to previous operation			
grade 3	3 (2.1%)	2 (1.6%)	
grade 4	14 (9.9%)	4 (3.2%)	0.07
Pelvic endometriosis			
grade 3	16 (11.3%)	7 (5.6%)	
grade 4	19 (13.5%)	30 (24.2%)	
Cooperated with pelvic peritonectomy	26 (18.4%)	27 (21.8%)	0.61
Complication			0.03
Transfusion	9 (6.4%)	19 (15.3%)	
Bladder injury	0	0	
Bowel injury	0	1 (0.8%)	
Postoperative fever	0	4 (1.6%)	
Intraperitoneal hematoma	0	0	
Reoperation	0	0	
Postoperative hospital stay			0.47
≤3 days	139 (98.6%)	121 (97.6%)	
6–8 days	1 (0.7%)	2 (1.6%)	
9–12 days	1 (0.7%)	0	
≥13 days	0	0	

**Table 4:** Comparison of postoperative states according by single-site laparoscopic fundusectomy vs. adenomyomectomy.

Clinical Outcome	Mean ± SD, No of Patients (%)		
	Fundusectomy	Adenomyomectomy	*p*-Value
Preoperative longitudinal length of uterus (cm)	10.04 ± 1.90	10.32 ± 10.56	0.76
Preoperative volume of uterus (gm)	332.55 ± 193.97	263.09 ± 144.53	0.00
Preoperative CA125 (U/mL)	68.37 ± 70.26	119.55 ± 170.03	0.00
Postoperative state 1 Mo later			
Longitudinal length of uterus (cm)	7.02 ± 1.02	8.51 ± 4.41	0.00
Weight of uterus (gm)	83.09 ± 40.20	136.48 ± 75.76	0.00
CA125 (U/mL)	19.73 ± 14.49	63.56 ± 62.06	0.00
Postoperative state 3 Mo later			
Longitudinal length of uterus (cm)	6.61 ± 0.97	7.26 ± 1.28	0.00
Volume of uterus (gm)	72.09 ± 32.32	94.45 ± 66.12	0.00
CA125 (U/mL)	16.19 ± 11.50	19.63 ± 19.68	0.41
Postoperative state 6 Mo later			
Longitudinal length of uterus (cm)	6.49 ± 1.04	7.58 ± 1.64	0.00
Volume of uterus (gm)	64.54 ± 42.36	108.52 ± 79.84	0.00
CA125 (U/mL)	14.42 ± 9.09	22.49 ± 20.58	0.00
Postoperative state 12 Mo later			
Longitudinal length of uterus (cm)	6.61 ± 1.18	7.73 ± 1.31	0.00
Volume of uterus (gm)	63.27 ± 35.39	109.95 ± 78.97	0.00
CA125 (U/mL)	17.16 ± 21.91	25.36 ± 20.87	0.06
Postoperative state 24 Mo later			
Longitudinal length of uterus (cm)	6.38 ± 1.09	7.80 ±1.47	0.00
Volume of uterus (gm)	58.11 ± 31.12	128.55 ± 143.16	0.00
CA125 (U/mL)	20.69 ± 22.07	55.02 ± 122.45	0.14
Reoperation	0	8 (6.5%)	0.00
Hysterectomy	0	4 (50%)	
Fundusectomy	0	4 (50%)	
Long term complication	0	0	

## Data Availability

The raw data supporting the conclusions of this article will be made available by the authors upon request and can only be shared anonymously.

## Abbreviations

The following abbreviations are used in this manuscript:

OC	Oral contraceptives
LNG-IUS	Levonorgestrel releasing intrauterine system
UAE	Uterine artery embolization
D&C	Dilatation and curettage
CS	Cesarean section
HIFU	High-intensity focused ultrasound
NSAID	Non-steroidal anti-inflammatory drug
